# Spatial Transcriptomics to Study Virus-Host Interactions

**DOI:** 10.1146/annurev-virology-092623-104926

**Published:** 2025-06-16

**Authors:** Chase Holdener, Shaowen Jiang, Danica M. Sutherland, Kira A. Griswold, Terence S. Dermody, John S.L. Parker, Iwijn De Vlaminck

**Affiliations:** 1 Meinig School of Biomedical Engineering, Cornell University, Ithaca, New York, USA; 2 Computational Biology Department, Cornell University, Ithaca, New York, USA; 3 Department of Pediatrics, University of Pittsburgh School of Medicine, Pittsburgh, Pennsylvania, USA; 4 Institute of Infection, Inflammation, and Immunity, UPMC Children’s Hospital of Pittsburgh, Pittsburgh, Pennsylvania, USA; 5 Department of Microbiology and Molecular Genetics, University of Pittsburgh School of Medicine, Pittsburgh, Pennsylvania, USA; 6 Baker Institute for Animal Health, College of Veterinary Medicine, Cornell University, Ithaca, New York, USA

**Keywords:** spatial transcriptomics, RNA sequencing, viral pathogenesis, viral tropism, viral immunology

## Abstract

The morbidity and mortality associated with viral diseases in plants, animals, and humans are significant concerns. Understanding how viruses cause disease and identifying the viral and host factors that determine the outcome of infection are essential to develop new antiviral therapeutics and strategies to induce protective immunity. In this review, we focus on the transformative potential of spatial transcriptomics for studies of viral pathogenesis and some of the intricacies of corresponding technologies and how to implement them.

## INTRODUCTION

The processes of infection and disease that take place in a virus-infected host are complex and diverse. Within an infected organ, there is usually a mixture of infected and uninfected cells. Among infected cells, there is even more diversity. Viral infection alters cellular physiology dramatically, and the response to infection is heterogeneous, depending on the timing of infection, the life cycle of the virus, and the host’s innate and adaptive immune responses. Immune signaling by host cells can alter and recruit diverse immune cell populations into the tissue to resolve infection, and the interactions between immune cells and infected and uninfected cells are spatially heterogeneous. Given this heterogeneity, the spread of viruses in tissues and the mechanisms by which viruses cause disease are difficult to quantify and study.

Approaches to investigate host responses to viral infection in vivo have relied mainly on gross pathology, histopathology, and immunohistopathology to define the distribution of molecules and cell types in infected tissues or flow cytometry to monitor changes in the activity, numbers, and distribution of immune cells. Genetically deficient animals and targeted ablation of immune cells or immune responses can be used to determine functions of particular genes or immune cell types in viral pathogenesis (1, 2). Discovery-based approaches to uncover genes that are differentially regulated during viral infection have primarily relied on bulk approaches such as RNA sequencing (RNA-seq), which cannot identify infected cell types or distinguish the responses of infected and uninfected cells (3). High-throughput single-cell RNA sequencing (scRNA-seq) and related technologies have been used to study viral infection and the underlying host responses at single-cell resolution (4–6). These approaches have uncovered the heterogeneity of viral infection at the level of individual cells (7). Nonetheless, while single-cell sequencing can identify infected cell types and cellular responses to infection, this technique does not preserve information about the spatial distribution of these responses or provide insights into the network of cell-to-cell signaling in tissues.

This review explores advances made possible by spatial transcriptomics (ST) in studies of viral pathogenesis. Guided by successful examples (8, 9) and our experience using different strategies, we discuss how spatial profiling of virus infection across organs and tissues can provide new insights into mechanisms by which viruses spread in complex organisms; the spatial and single-cell heterogeneity of innate immune responses; the causes of local tissue injury due to infection; and the heterogeneous interactions between immune, infected, and bystander cells in the context of complex tissues. We discuss technical challenges and anticipated solutions and provide a forward-looking perspective on the trajectory of viral pathogenesis studies using ST.

## WHAT CAN BE LEARNED ABOUT VIRAL PATHOGENESIS FROM SPATIAL TRANSCRIPTOMICS APPROACHES?

Before discussing ST technology for studies of viral pathogenesis, we consider what can be learned using ST that cannot be readily evaluated using other approaches. Traditional approaches such as histology and immunohistochemistry can identify infected organs, the degree of tissue damage, immune and inflammatory responses, and, to some degree, the types of cells that are infected or are responding to infection. However, these approaches can miss cell types that are rarely infected and may not detect early activation or infiltration of immune cells. Moreover, these approaches are limited to a select number of targeted proteins, which capture only part of the complexity of the tissue. In contrast, unbiased host transcriptomic signatures, together with the detection of viral transcripts, are a new way to identify infected cells, including cells that may not otherwise be marked as infected. In addition, ST data can be used to define responses of these cells to infection and further subclassify them.

**Figure 1:**
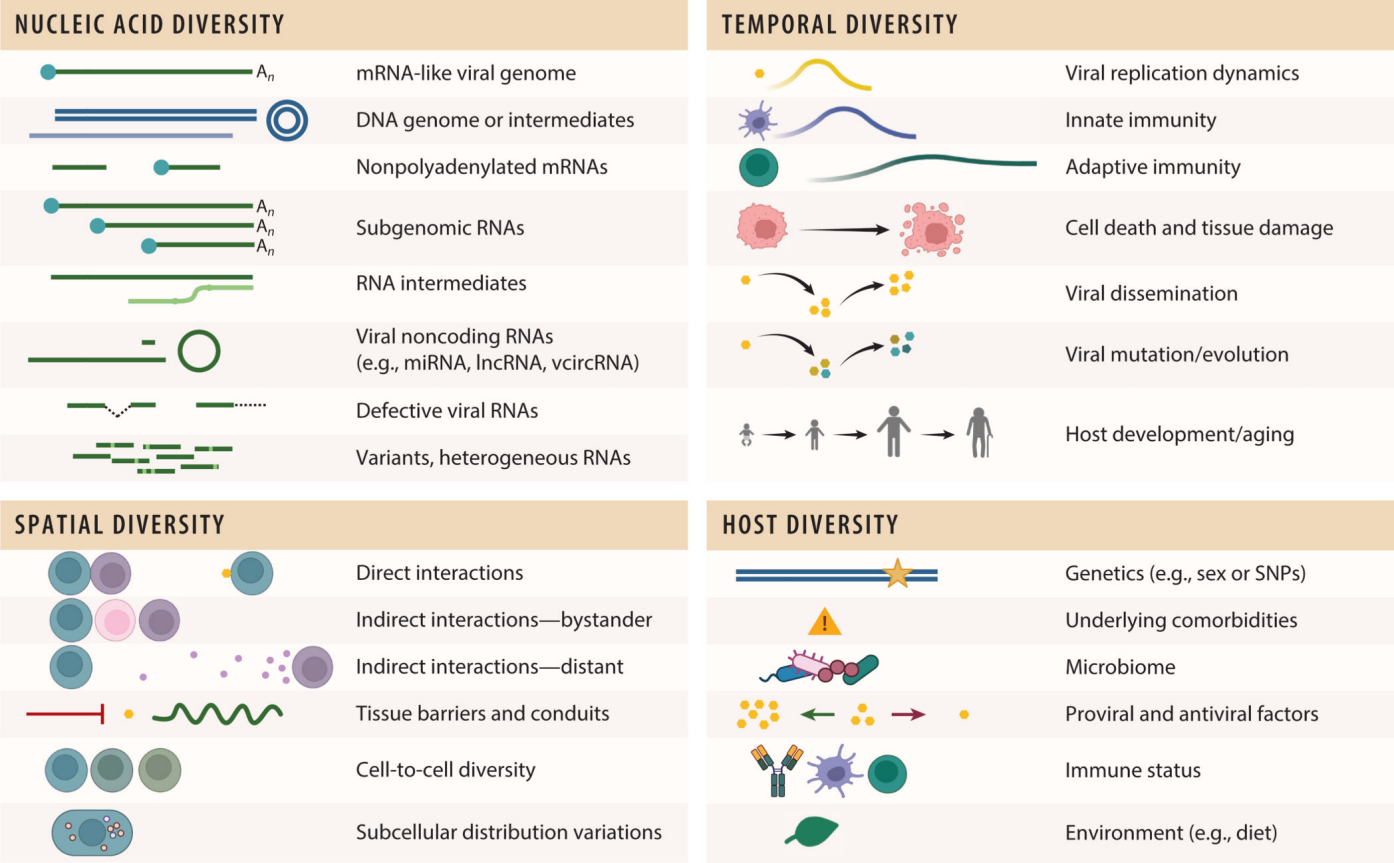
A summary of key considerations and challenges in viral and host diversity when planning and conducting ST studies. Figure adapted from images created in BioRender; Sutherland D. 2025. https://BioRender.com/m55b486. Abbreviations: lncRNA, long noncoding RNA; miRNA, microRNA; mRNA, messenger RNA; SNP, single nucleotide polymorphism; ST, spatial transcriptomics; vcircRNA, circular RNA derived from virus.

A common theme emerging from transcriptomic studies is the heterogeneity of host infection responses (10–13). Spatial heterogeneity and its biological relevance raise exciting questions that can be answered using ST (Figure 1). Fine details of the spatial-temporal distribution of infected cells, particularly their location relative to neurons and vasculature, can provide insight into the spread of viruses through tissues. An important aspect of the host response to infection that is challenging to appreciate using conventional approaches is the extent of the bystander cell response to viral infection. Interferons and other cytokines affect gene expression and hence cellular transcriptomes, particularly in vascular and immune cells (14, 15). These responses and their spatial distribution can provide clues about the nature and extent of the host immune response to viral infection in a tissue. The bystander responses of different cell types surrounding foci of infection also can be quantified and compared. Because ST data can be used to classify different cell types, they can provide information about the temporal changes in cell-type distribution during viral infection. For example, ST analyses can identify the types and numbers of inflammatory and immune cells that infiltrate into virus-infected tissues and determine the spatial distribution of these cells relative to virus-infected cells and damaged tissues. If ST experiments assess animals at different times post-inoculation, temporal changes in cell infiltration can be discerned. Another application of ST to viral pathogenesis research is to define the activation of immune and inflammatory signaling pathways in situ. Both the cells eliciting the signals and the cells in the vicinity responding to the signals can be identified, allowing contextual effects of the signaling to be defined. By combining ST with long-read sequencing, it is possible to identify viral variants and localize them in tissues.

## GENERAL CONSIDERATIONS WHEN USING SPATIAL TRANSCRIPTOMICS APPROACHES TO STUDY VIRAL PATHOGENESIS

Tracking viral infection in vivo using transcriptomic technologies requires detecting viral and host RNA in infected tissues. Each virus has unique properties (e.g., genome type, replicative efficiency, tropism, and biosafety considerations) that must be considered to allow efficient codetection of pathogen and host RNAs (Figure 1). The replicative strategy of a virus is an essential first consideration when planning ST studies. While many viruses produce abundant transcripts that are both capped and polyadenylated, others produce transcripts that are temporally regulated, are nonabundant, lack a cap, or, in some cases, lack a poly(A) tail. In addition, it is useful when studying viruses that contain an RNA genome to detect replicative RNA strands, as these indicate that a cell is productively infected. Depending on the nature of viral transcripts, the technology used to detect these RNAs may require modification to allow efficient capture and amplification. The detection of noncoding RNAs (ncRNAs), such as microRNAs (miRNAs) produced by DNA viruses and retroviruses, also may be of interest when designing studies.

### Not All Viral Messenger RNAs Are Polyadenylated

Most sequencing-based transcriptomic technologies capture transcripts by hybridization of the polyadenylate transcript tail to a substrate-bound poly(dT) oligonucleotide. However, viral messenger RNA (mRNA) and genomic RNAs may or may not be polyadenylated. Several viruses synthesize mRNAs that are not polyadenylated, including members of the *Arenaviridae*, *Bunyaviridae*, *Flaviviridae*, and *Reovirales* (16–18). Such nonpolyadenylated transcripts can be captured by modification of the capture technology to use either specific complementary sequences or enzymatic in situ polyadenylation of all RNAs before conventional poly(dT) capture (19, 20).

### Replicative Strands, Subgenomic RNAs, microRNAs, and Other Noncoding RNAs of Viruses Can Be Captured

Detection of replicative RNAs provides robust supporting data to indicate a cell is infected. Due to the nature of the poly(dT) capture technology used with many ST platforms, nonpolyadenylated RNAs can be captured at varying frequencies if they contain a series of consecutive adenine residues (8, 21). However, capture efficiency varies between RNAs, preventing accurate quantification. Complementary replicative RNA strands can be captured following in situ polyadenylation (19). Many RNA viruses also produce subgenomic mRNAs or nested RNAs. It may be possible to computationally distinguish such RNAs from genomic or replicative RNAs (22).

Other ncRNA transcripts expressed by viruses include long ncRNAs (lncRNAs), miRNAs, and circular RNAs derived from viruses (vcircRNAs) (23–25). These types of viral RNAs often function in modification of host gene expression and viral pathogenesis. For example, many DNA viruses and some enteroviruses, flaviviruses, retroviruses, and others synthesize viral miRNAs. Viral miRNAs serve essential functions in regulating latency, controlling host-cell proliferation and survival, and evading host immune responses. Some viruses produce ncRNAs that lack a poly(A) tail and dominate the transcriptome (26, 27). These include Epstein-Barr virus EBER ncRNAs and adenovirus-associated ncRNAs. Detecting these types of RNAs using ST will, in most cases, require protocol modification. We discovered that in situ polyadenylation can be used to polyadenylate viral and host RNAs (19), allowing the capture of lncRNAs, miRNAs, and other ncRNAs. This approach should enable detection of ncRNAs, low-abundance mRNA transcripts, and replicative strands for other RNA virus systems. However, sequencing of vcircRNAs usually involves depletion of linear RNAs (28) and, therefore, is not readily compatible with current ST approaches.

### Not All Cells Associated with Viral Transcripts Are Infected

An important consideration in ST that has reached cellular or subcellular resolution is whether the cell types identified as harboring viral transcripts are infected. mRNA released from damaged cells may adhere to the surface of some cell types, e.g., red blood cells (29). In addition, conventional phagocytic and antigen-presenting cells may phagocytose cellular material containing viral RNA but not be infected. Therefore, it is important to consider whether the cell types identified as harboring viral transcripts are truly infected or contain viral transcripts released from infected cells. These events can be excluded by either in vitro validation studies or setting a false-positive threshold to exclude cells with low levels of transcripts (30). This threshold can be established by comparison to transcript counts from known virus-susceptible cell types in a tissue or ambient RNA detected in control samples, similar to strategies used for scRNA-seq (31).

### Some Viruses May Induce Host-Cell Shutoff

Some virus-encoded proteins, such as herpes simplex virus ICP27 (32), can cause host-cell transcriptional shutoff following infection, limiting the transcriptional data that can be gathered from infected cells. However, the relative absence of host transcripts can identify virus-infected cells. Host responses of surrounding bystander cells also can help define areas in which cells are infected.

### Viral Strain Variations

A variety of exciting questions can be answered using ST by altering the infecting virus strain or strains. Mechanisms governing viral dissemination, competition, disease severity, and restriction are easily addressable with these discovery-based strategies. For example, comparing control tissues with those infected with low- and high-virulence strains can provide a dataset to define factors governing disease (33). RNA viruses generally have low genome replication fidelity, and infection may yield virus variants. Virus and host bottlenecks can select for dominance of certain virus strains over time or in tissues distant from the primary site of infection (34). Mapping how viral variants spread across tissues could be used to better understand viral dissemination in a host and how some viruses undergo extinction events whereas others experience expansion, allowing definition of how new viral variants disseminate and dominate. Similarly, sequencing data may reveal spatial rationale about factors influencing the prevalence or distribution of differential gene expression, defective viral sequences, and recombination or splicing events. Longer reads with higher sequencing depth may be necessary to accurately capture these minority events (35, 36) and spatially map viral variant dynamics across the tissue. If viruses do not substantially differ genetically, virus inocula can be genetically barcoded to probe viral population dynamics in vivo over time (37). ST also offers the opportunity to investigate coinfections to better understand how two or more pathogens, such as human immunodeficiency virus (HIV) and *Mycobacterium tuberculosis* (38), interact in discrete tissue niches to accelerate disease.

### Route of Inoculation and Viral Dose

The dose of virus often refers to the number of infectious virus units delivered to a host. It is generally reported in experimental settings but can be challenging to estimate in natural infections. The true viral inoculum is a heterogeneous mixture, components of which may alter replication and disease. For example, influenza pathogenesis is diminished when the inoculum is enriched with defective particles (39), and an immunosuppressive component of mosquito saliva coinoculated with Zika virus enhances viral replication (40). Thus, a dose should be carefully prepared and quantified. The viral dose should be based on a biologically relevant dose observable in natural infections whenever feasible. As an increased virus dose is often attributed to more severe disease (41), it may be valuable to compare spatial transcriptomes at multiple doses.

While many viruses generally have one route of transmission, some viruses can use several routes to enter a host and cause disease. The route of inoculation, such as intravenous, intra-gastric, or intranasal, also can alter viral replication, dissemination, and pathogenesis (42). Even similar inoculation strategies to target the lung can produce disparate results (43, 44). In some circumstances, exploring pathogenesis using a route of inoculation not used in nature may be informative. For example, in experimental neurovirulence studies, neurotropic viruses are often inoculated intracranially to bypass dissemination and virulence at other body sites (41, 45). A virus dose useful for one route of inoculation may be inefficient for another (46), and thus, virus dose and route of inoculation should be coordinated.

### Kinetics of Viral Infection in Animals

Viral spread from the site of inoculation and the kinetics of infection must be considered in determining when tissue samples should be collected for ST. Collecting samples when peak viral replication occurs may not be as informative as collecting samples earlier or when the virus first reaches secondary replication sites. Basic knowledge of the routes of spread, the timing of infection of different tissues, and the degree of tissue damage will inform the optimal timing and tissues to collect for ST. One important use of ST is examining tissues over time to assess viral spread, comparing the cell types infected early and late during infection, and defining the types of immune and inflammatory cells that infiltrate the tissue (8, 47). Additionally, studies of chronic stages of infection (48) or even post-clearance of virus, when immune sequelae dominate the disease (49), can provide new insights about viral illness following the acute phase of viral infection. A particularly important application of ST is the detection of viral integrations into host DNA (50), which could be used to identify reservoirs of latency, such as with HIV, and to characterize biomarkers or spatial features of these important cellular niches.

### What Organs and Tissues Should Be Collected?

ST is, in principle, amenable to any tissue that can be sectioned, including decalcified bone (51). A challenge for many ST platforms is the limitation on tissue size that can be analyzed. ST platforms are currently limited to 2D tissue slices (∼10- to 20-μm thick), and sequencing-based platforms are further limited by the size of the tile for RNA collection (e.g., a tile of 1 cm^2^). While 3D tissue reconstruction is possible with computational alignment of parallel 2D slices (52, 53), these experiments are more expensive and may miss information between parallel slices. In some cases, a given tissue should be divided to allow assay by multiple techniques. For example, evaluating hepatotropic virus infection by processing one liver lobe for scRNA-seq and another for ST would be a powerful complementary approach. Additionally, tissues can be stored at −80°C or in RNase-free solutions while conducting validation studies to confirm RNA quality or the presence of viral infection using other strategies such as immunohistochemistry or viral load determination. The tissue block used to prepare spatial slices also can be rebanked for later validation studies of gene candidates identified with ST. Another consideration is whether to focus on sites of viral infection or disease. Infections by some viruses cause disease affecting tissues where viral replication does not occur, e.g., autoimmune-mediated diseases (54).

### Biosafety Considerations

Attention to biosafety is essential but can complicate some viral pathogenesis experiments or clinical sample collection by imposing additional biocontainment measures (55). As specific policies, practices, and equipment may differ among institutions, we recommend designing ST experiments in coordination with biosafety officers. For biosafety level (BSL)-2, BSL-3, and BSL-4 viruses, appropriate laboratory techniques must be considered to (*a*) inoculate animals, (*b*) obtain tissue, and (*c*) prepare tissue sections for imaging or sequencing. An advantage of ST over single-cell transcriptomics is the relatively limited manipulation of infected tissue, as extended sample manipulation can cause cell stress and hypoxia and contribute to transcriptomic artifacts (56). Reduced sample manipulation is desirable for studies of virulent viruses and clinical samples, where processing time is constrained.

As with other transcriptomics approaches, the success of ST relies on preserving RNA integrity and abundance. Therefore, many strategies recommend using fresh or fresh-frozen tissue, for which a cryostat must be available in the containment facility. Additionally, virus must be in-activated using formalin fixation, heat, or other chemicals before removal from high-containment settings, as has been done for studies of hepatitis B virus (50) and West Nile virus (57). As fresh-frozen tissue may not be available, for example, in studying postmortem severe acute respiratory syndrome coronavirus 2 (SARS-CoV-2) patient samples (58), many platforms incorporate options for formalin-fixed paraffin-embedded (FFPE) tissue. FFPE tissues should be prepared following the manufacturer’s guidelines to minimize degradation and cross-linking of RNAs. Informative studies also may be conducted using attenuated BSL-2 strains of otherwise BSL-3 viruses.

## PLATFORMS AND TECHNIQUES

**Figure 2:**
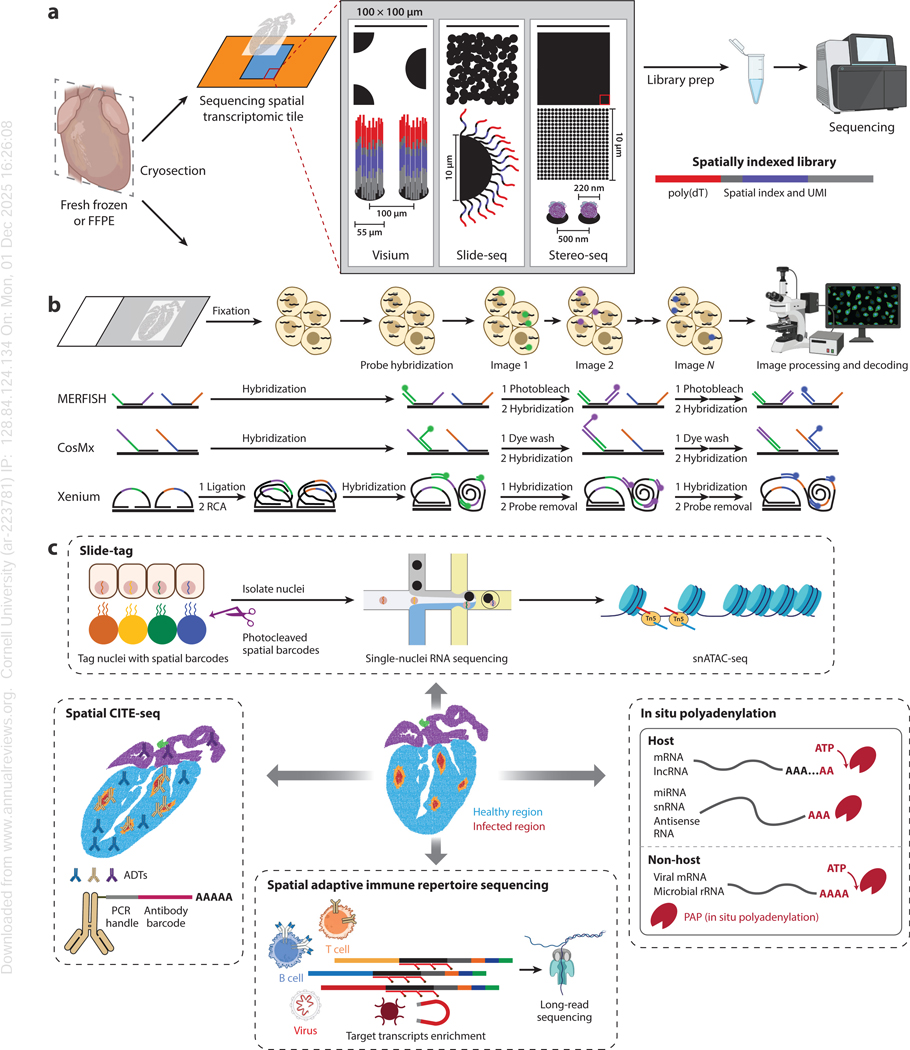
Overview of ST workflows and various advanced methods for multi-omics applications. (*a*) Schematic of the experimental workflow for sequencing-based approaches, including Visium, Slide-seq, and Stereo-seq. (*b*) Schematic of the experimental workflow for imaging-based approaches, including MERFISH, CosMx, and Xenium. (*c*) Diagram of different advanced methods that can be applied to ST techniques, including Slide-tag, Spatial CITE-seq, in situ polyadenylation, and Spatial AIRR. Abbreviations: FFPE, formalin-fixed paraffin-embedded; lncRNA, long noncoding RNA; miRNA, microRNA; mRNA, messenger RNA; rRNA, ribosomal RNA; snATAC-seq, single-nucleus assay for transposase-accessible chromatin sequencing; snRNA, small nuclear RNA; ST, spatial transcriptomics; vcircRNA, circular RNA derived from virus. Figure adapted from images created with BioRender.com; Jiang S. 2025. https://BioRender.com/tad1lxl.

ST methods can be divided into two major classes: sequencing based and imaging based. The selected method dictates many aspects of the experiment, including costs. In this section, we explore the merits, challenges, and opportunities associated with each of these classes (Figure 2). We also highlight emerging methodologies that improve performance or extend the range of measurements that can be pursued. Several reviews discussing ST methodologies (59–63) are informative. Here, we highlight key concepts and discuss applications of these methods to study viral tropism and virus-host interactions.

### Sequencing-Based Spatial Transcriptomics

In sequencing-based ST, RNA from a tissue section is transferred to a spatially indexed surface, allowing the tissue location of the RNA to be inferred from sequencing results (64). The principle of spatially resolved RNA sequencing was first demonstrated in 2016 by Ståhl et al. (65), who used microarray spotting to engineer an array of spatially barcoded DNA oligonucleotides. The DNA oligonucleotides contained a short DNA sequence specific to the location or pixel and a poly(dT) sequence at the 3′ end that could hybridize with poly(A)-tailed mRNA. Reverse transcription yielded a complementary DNA (cDNA) copy of the original mRNA tagged with the DNA barcode. This initial work achieved only modest spatial resolution, with spot diameters of 100 μm and spot center-to-center distances of 200 μm, but demonstrated a scalable principle. A commercial version of this technology was released in 2019 by 10x Genomics, with spot sizes of 55 μm and spot center-to-center distances of 100 μm (Figure 2a). Visium-HD by 10x Genomics achieves higher resolution with an array of 2 × 2 μm barcoded squares without gaps in tissue coverage (66). However, unlike other platforms described in this section, Visium-HD is probe based and species limited and requires preparation and integration of virus-specific probes. Slide-seq, now offered by Curio Bioscience, uses DNA-barcoded beads with a 10-μm diameter (64). These beads are spatially indexed and densely packed on a tile surface. On-bead conversion to cDNA records the spatial DNA barcode and pixel size (10 μm), enabling near-single-cell resolution. Stereo-seq, offered by Complete Genomics Incorporated, uses a dense array of barcoded DNA nanospheres, each measuring ∼220 nm with a center-to-center distance of 500 nm, to achieve subcellular spatial resolution (67). Seq-Scope, reported in 2021 by Cho et al. (68), leverages the Illumina sequencing platform to enable subcellular resolution by solid-phase amplification of randomly barcoded DNA oligos and spatial barcodes. Seq-Scope achieves a center-to-center resolution of ∼600 nm. A related method, Open-ST, achieves a spot resolution of ∼600 nm and a center-to-center resolution of ∼600 nm (69).

In addition to improvements in spatial resolution, continuous enhancements have been made to the capture area and the sensitivity and versatility of spatial RNA-sequencing methods. A larger capture area provides a larger field of view, which is important for whole-organ and whole-animal ST (70). Commercial and customized methods now achieve capture areas of 10 × 10 mm. The sensitivity determines the number of molecules detected per unit of surface area. This sensitivity depends on several factors, including sequencing depth and the efficiencies of recovery and reverse transcription of mRNA. Initially, spatial RNA sequencing was only possible using fresh-frozen tissues, but FFPE tissues now can be assayed, which allows analysis of archival samples (71). For sequencing-based ST methods, costs are defined by the components, reagents, and DNA sequencing, which depend on sequencing depth and platform used.

### Imaging-Based Spatial Transcriptomics

Imaging-based ST uses in situ hybridization to visualize nucleic acids in tissues. In 2008, Raj et al. (72) used fluorescence in situ hybridization (FISH) to detect individual mRNA molecules in cells. While complexity was limited to just three mRNA species in this early work, it identified a critical engineering principle. In subsequent years, combinatorial multiplexing and sequential imaging techniques have been developed to enhance the repertoire of RNAs measured (73–75). Transcript molecules are decoded through multiple imaging rounds using various coding schemes and multiple fluorescent channels (76–78). For instance, MERFISH, now commercialized by Vizgen, uses error-robust binary encoding (78). CosMx by Nanostring employs a limited number of probes amplified via branch chain hybridization (79), while Xenium from 10x Genomics uses targeted padlock probes and rolling-circle amplification (80) (Figure 2b). Systematic bench-marking studies have compared these platforms and showed good concordance with orthogonal RNA-seq datasets, validating their ability to capture biologically relevant signals (81). In situ sequencing, including STARmap developed by Wang et al. (82), can be used to detect in situ amplified nucleotide sequences in cells via sequencing by ligation. Imaging-based ST offers high spatial resolution and high sensitivity, making it attractive for studying viral pathogenesis. However, there are limitations. Compared with sequencing-based methods, imaging approaches measure fewer transcript types, contributing to inherent data bias. While custom panels can be designed to detect viral RNA, predefined gene panels are used, limiting discovery-driven studies. Also, these methods can be time-consuming and labor-intensive, given that multiple rounds of hybridization, washing, and imaging steps are needed for most protocols. Moreover, imaging methods primarily measure transcript abundance and do not capture sequence variation, which is essential for identifying viral variants or differential RNA processing. For imaging-based ST methods, the most important costs are associated with instruments and reagents.

### Advanced Methodologies

The development of ST platforms with single-cell resolution capacity represents a significant breakthrough. Curio Trekker was the first commercially available ST platform with true single-cell resolution (83). However, this resolution comes at the cost of increased assay complexity and spatially sparser data. Nonetheless, spatial information is retained while providing nucleotide-level insights, making them valuable for studying viral variants and virus-host interactions. Advanced spatial methodologies integrate transcriptomics with additional information (84), enabling a more comprehensive view of tissue biology. Spatially resolved assays for chromatin profiling and DNA sequencing allow mapping of chromatin accessibility and genomic features, which will be useful to study changes in gene regulation induced by viral infection (85, 86). DBiT-Seq, developed by Liu et al. (87), uses microfluidics for deterministic barcoding of nucleic acids in tissues. This strategy can simultaneously identify and map proteins and mRNAs. Spatial CITE-seq extended the multiplexity in protein detection to 200–300 proteins (88), allowing robust spatial analysis of both RNA and protein in a tissue section. SPOTS incorporated antibody-derived tags within Visium to profile proteomics with more than 30 protein markers (89). Innovations in capturing nonpolyadenylated transcripts (19), including viral and microbiome-derived RNAs (20, 90–92), provide a more complete view of RNA populations. Patho-DBiT enables spatial whole transcriptome sequencing in clinical FFPE tissues (93). These methods make it possible to profile RNA viruses and detect replicative intermediates. Other advances have allowed the spatial mapping of B and T cell immune repertoires (94–97), which are central to understanding host immune responses to viral infections. These methods allow sequences of immune receptor genes (e.g., B and T cell receptors) to be determined while preserving spatial information, providing insights into clonal expansion, immune cell localization, and tissue-specific adaptive immune responses (Figure 2c).

## COMPUTATIONAL CHALLENGES AND SOLUTIONS

**Figure 3:**
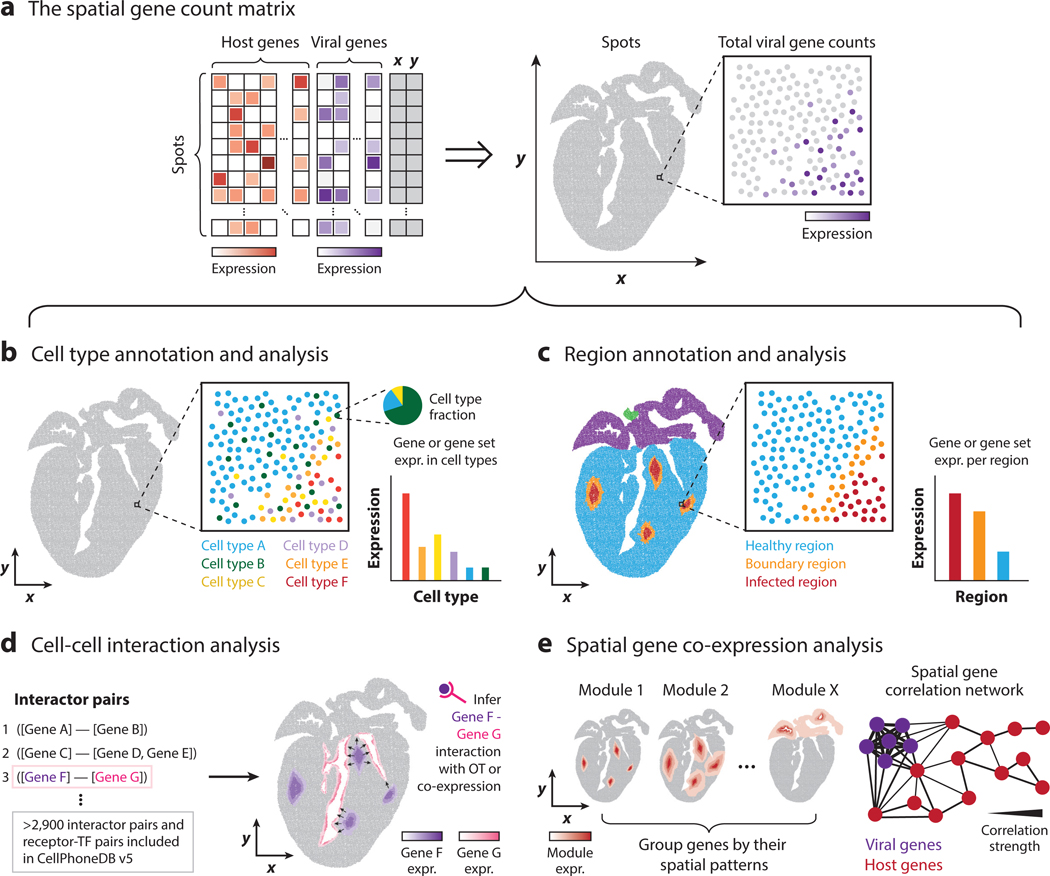
Overview of the spatial RNA-seq gene count matrix and various subsequent data analysis approaches. (*a*) The spatial gene count matrix contains spatially resolved transcriptomes, where each transcriptome has an (*x*,*y*) coordinate. Virus transcripts (or any transcripts of interest) can be located in the tissue space. (*b*) Deconvolution algorithms allow prediction of cell-type makeup for each spatial location using a reference scRNA-seq dataset. (*c*) Various tissue segmentation algorithms allow the division of tissue into regions, using both the gene expression profiles and spatial locations of the transcriptomes. (*d*) Cell-cell interaction analysis evaluates interactor pairs or signaling pathways in their spatial distribution. Spatially co-expressed or nearby interactors can be quantified using OT or co-expression approaches. (*e*) Spatial co-expression analyses group genes by their spatial expression patterns, revealing complex gene programs. Constructing networks of spatial gene correlation can identify and order host genes that are associated with viral genes. Abbreviations: Expr., expression; OT, optimal transport; RNA-seq, RNA sequencing; scRNA-seq, single-cell RNA sequencing; TF, transcription factor.

A single ST experiment can produce hundreds of thousands of spatially resolved transcriptomes. There is almost certainly interesting biology in each dataset, but it can be challenging to analyze such large amounts of data. This section describes the ST count matrix data structure and describes spatial analyses, including cell-type mapping, tissue region annotation, cell-cell interaction analysis, spatial gene correlation analysis, and multi-dataset integration approaches. In virology, these analyses can (*a*) reveal sites of viral infection; (*b*) discern cell types, cell interactions, and genes important for viral infection; (*c*) spatially and temporally order the immune response cascade; and (*d*) distinguish immune response and tropism-guiding host genes, among other applications (Figure 3).

### Spatial RNA Sequencing Compared to scRNA Sequencing

The output of ST data is a gene count matrix, with transcriptomes represented along one axis and counts for individual genes along the other. These data resemble scRNA-seq data structures except for two major differences. First, each transcriptome has a unique (*x*,*y*) spatial coordinate in the tissue sample, and second, each transcriptome is not necessarily a single cell, as it may contain RNA from neighboring cells or the extracellular matrix (Figure 3a). The number of cells contributing to each spatial transcriptome depends on the spatial resolution of the platform. For example, Visium has spot diameters of 55 μm, capturing RNA from 10 or more cells per spot (65). In contrast, Slide-seq uses 10-μm beads, enabling higher resolution by capturing RNA from only a few neighboring cells (64, 98). Emerging high-resolution sequence-based platforms, such as Stereo-seq and OpenST, and imaging-based platforms offer submicron resolution (67, 69, 78), providing new opportunities and challenges. A critical task is grouping multiple spots into coherent cells, which is often achieved using paired conventional histology to delineate nuclei and approximate cell boundaries or binning approaches that combine spots with a user-specified spatial grid. Advanced cell segmentation algorithms, such as Baysor and SCS, also can be used for subcellular resolution data (99, 100). Subcellular spatial resolution does not inherently translate to single-cell resolution, as diffusion of RNA and other sources of noise are present, and cell segmentation methods are imperfect. These limitations influence how spatial data are analyzed.

### Assigning Cell Types and Regions to Spatial Transcriptomes

Examining how cell types are spatially distributed in tissues is one of the most potent analyses enabled by ST. However, because ST does not achieve true single-cell resolution, standard scRNA-seq dimension reduction and cell-type clustering approaches are less effective when applied to spatial data. Deconvolution algorithms, such as cell2location (101), BayesPrism (102), and CARD (103), overcome this limitation. These algorithms use a reference scRNA-seq dataset to predict the cell-type composition of each spatial transcriptome. After deconvolution, each transcriptome is labeled with a cell-type score for all cell types in the single-cell reference, allowing assignment of each transcriptome to the cell type with the highest score (Figure 3b). The need for a single-cell RNA reference to use as input along with spatial data can be a challenge. However, there are many single-cell databases and atlases available for human, mouse, and other species across tissue types, including the Allen Brain Cell Atlas (104), HuBMAP (105), Human Cell Atlas (106), Mouse Cell Atlas (107), *Tabula Muris* (108), Tabula Sapiens (109), and more.

Spatial transcriptomes also can be clustered according to tissue regions or cellular neighborhoods (Figure 3c). Tissue regions are usually less granular than cell-type patterns. Identifying these regions involves finding shared gene expression profiles that span multiple neighboring transcriptomes. Regions may reflect heterogeneous tissue compositions, injury zones, or boundary injury zones. Many tissue region identification algorithms can assist with this task, including BayesSpace (110), GraphST (111), SEDR (112), SpaGCN (113), and STAGATE (114). Tissue region identification reveals the underlying structure of the dataset and allows subsequent analyses. For example, differential gene expression analysis can be conducted to identify regional gene differences. In this approach, upregulated or downregulated genes are identified in a particular region, such as sites of infection or inflammation. These differentially expressed genes are likely involved in the immune response or influence viral tropism. Furthermore, proximity analyses can be conducted with labeled regions to define gene expression or cell-type abundance changes along a spatial axis in tissue.

### Cell-Cell Communication Analysis

Cell-cell interactions are central to antiviral immune responses, allowing cells to communicate and alter gene expression. These interactions occur by either direct contact between cell membrane proteins of neighboring cells or ligand-receptor signaling, which can mediate communication over short or long distances. Numerous databases catalog cell-cell interaction data, including CellChatDB (115) and CellPhoneDB (116). These resources provide curated lists of interactor pairs, where each pair can consist of individual proteins or ligands or complexes of multiple proteins or ligands. CellChatDB and CellPhoneDB provide R and Python packages for scRNA-seq data to deduce enrichment of interaction pairs across cell types and can be used to infer cell communication. ST provides a stronger context for cell-cell interaction analysis than single-cell data, as it incorporates physical distance between cells to evaluate interactor pairs (Figure 3d). This spatial dimension is valuable in infection studies, where proximity often influences communication dynamics. COMMOT uses optimal transport theory to compare spatial distribution of interactor pairs in a specified distance constraint and generates visualizations of inferred signaling directionality (117). SpaTalk, SpatialDM, and other methods find spatial co-expression of ligands and receptors to infer cell communication (118–120). While the ST field is advancing cell-cell communication analysis, the scRNA-seq field currently provides more examples of cell-cell interaction analysis (121) and associated methods (122).

### Spatial Gene Correlation Analysis

Another strategy to analyze the spatial gene count matrix involves grouping genes into modules based on spatial expression patterns across spots (Figure 3e). Instead of grouping each spatial transcriptome based on all gene values, genes are grouped by expression pattern across all spatial transcriptomes. Spatial gene co-expression is a powerful approach for viral infection studies, as host genes or gene programs that are co-expressed with viral genes or any genes of interest can be identified. These virus-correlated host genes may be important for immune responses or viral tropism.

There are several different spatial gene correlation methods available. Hotspot groups genes into modules using a local spatial correlation test statistic and hierarchical clustering (123). Smoothie uses a computer-vision-inspired Gaussian smoothing technique to address noise and sparsity in the data and then constructs a spatial gene correlation network that positions and clusters genes into modules (124). The network output also may reveal spread of the host immune response at the gene level (Figure 3e). STAMP uses a simplified graph convolution network and topic modeling to split spatial transcriptomes into regions of similar gene expression and then identifies spatial pattern modules from these regions (125). In contrast to Hotspot and Smoothie, STAMP uses a GPU. Other methods examined spatial gene correlations in Visium ST data (126, 127). However, these methods do not efficiently scale to larger datasets produced by newer ST platforms.

### Integrated Analysis of Data from Healthy and Infected Tissues

An uninfected control dataset allows comparison of the cell-type distribution, cell-communication activity, and gene expression distributions with an infected sample. While there are likely uninfected cells in any infected tissue slice, having a true uninfected control sample strengthens these analyses and opens new approaches. First, cell-type positioning differences in uninfected and infected tissues can be examined. For example, the average distance between each cell type pair in uninfected and infected tissues can be compared. This analysis may reveal cell types that spatially colocalize in the infected tissue, suggesting virus-dependent cell-cell interactions. Changes in cell communication between healthy and infected tissues can be investigated using cell-communication analysis methods (117–120). It also is possible to investigate host tropism using these datasets. For this analysis, the first step is identifying host genes in the infected sample that are spatially correlated with viral genes using an analysis method such as Smoothie (124). The next step is filtering for genes that have similar expression patterns in both uninfected and infected samples, as these genes are consistent regardless of infection status and thus may guide where the virus disseminates. To find such genes with consistent patterns, Smoothie offers a multi-sample analysis in which all virus-correlated host genes can be ranked from the most stable spatial pattern to the least stable spatial pattern across the two samples. The most stable genes comprise the pool of candidate tropism-guiding host genes, which can be narrowed to cell membrane–associated proteins or other candidate genes. The final candidate list can be screened in silico with AlphaFold 3.0 (128) to predict protein-protein interactions (PPIs) between viral attachment proteins and candidate tropism-guiding host proteins. Top predicted interacting host proteins then can be validated in vitro. Combining spatial gene correlation, deep learning structural PPI prediction, and experimental validation provides a powerful approach to predict host genes that influence viral entry into cells and other steps in viral replication.

## PERSPECTIVE

Multiplexed profiling of virus-host interactions using ST is a relatively new approach for studying viral infection and disease in host tissues (Table 1). For example, the technology has been used to define cellular niches of viruses (8, 129–131), understand mechanisms promoting cancer by oncogenic viruses (132, 133), characterize host immune responses (8, 49, 57, 130, 134, 135), illuminate spatiotemporal roadmaps of viral dissemination and disease (8), and contribute to the design of antiviral therapeutics (136). However, significant barriers to widespread adoption must be overcome before the full potential of ST can be realized. Among these barriers are the current high cost of ST assays and the complexity of experimental procedures and data analysis. These factors slow the adoption of ST by new users and limit the scope and pace of discovery. We anticipate that ongoing advances in technology will overcome these challenges. Innovations in software and artificial intelligence promise to streamline and simplify the analysis process, especially for investigators with limited bioinformatics experience. At the same time, continued improvements in assay robustness and versatility will increase the accessibility and reliability of ST and facilitate broader adoption. Given the rapid pace of technological advancement, we expect the resolution, multiplexity, and sensitivity of ST methods to improve. Beyond transcriptomics alone, which was the focus of this review, integrating ST with other omics approaches—such as spatially resolved proteomics, chromatin accessibility, and metabolomics—will provide a more comprehensive view of virus-host interactions. Thus, ST is not merely a method to analyze where and how viruses influence tissues but potentially a foundational technology for the future of virology and immunology research. With the anticipated reduction in sequencing and instrument costs, achieving a complete 3D ST atlas of an entire organ will become possible. As ST continues to evolve, it will substantially change our approach to studies of viral disease and yield unanticipated antiviral strategies.

## Figures and Tables

**Table 1:** Utility of ST for studies of viral pathogenesis

Virus(es)	Method)s_	Key findings	Reference
Adeno-associated viral vectors	USeqFISH	Developed USeqFISH approach to enable high-resolution ST for endogenous and viral RNA profiling in intact tissues	Jang et al. 2023 (137)
	STARmap PLUS	Spatially mapped the transduction patterns of adeno-associated virus to delineate tissue- and cell-type-specific transduction landscapes at single-cell resolution across the mouse central nervous system	Shi et al. 2023 (138)
Dengue virus	Visium	Investigated spatial distribution of host response gene expression in liver tissues from mice infected with dengue virus	Chen et al. 2024 (33)
Reovirus	Visium; Slide-seq	Generated a high-resolution, spatially resolved transcriptome map of viral myocarditis in neonatal mice, revealing spatially distinct cellular phenotypes, cell-cell interactions, and immune-mediated injury responses	Mantri et al. 2022 (8)
	Visium + STRS	Developed an STRS approach, which enables detection of nonpolyadenylated viral RNA transcripts, revealing their spatial distribution and host responses during infection	McKellar et al. 2023 (19)
SARS-CoV-2	RNAScope; Nanostring GeoMX	Profiled ST atlas in regions of interest across lung tissues from multiple individuals, showing spatially heterogeneous inflammatory activation in different regions	Delorey et al. 2021 (139)
	Visium	Generated an ST map of SARS-CoV-2-infected placentae using human fresh frozen sections, revealing distinct proinflammatory niches related with high SARS-CoV-2 transcripts	Barrozo et al. 2023 (130)
	Visium; Nanostring GeoMX and CODEX; RNAScope	Developed a spatial multi-omics platform to investigate SARS-CoV-2-infected lung tissues, demonstrating spatial heterogeneity in expression related to cytokine signaling and interferon responses	Tan et al. 2023 (140)
Zika virus	Visium	Identified distinct Zika virus–associated placental niches characterized by upregulated immune and complement cascade components, localized to macrophage-rich microenvironments	Barrozo et al. 2024 (129)

Abbreviations: SARS-CoV-2, severe acute respiratory syndrome coronavirus 2; ST, spatial transcriptomics; STRS, spatial total RNA sequencing; USeqFISH, ultrasensitive, sequential fluorescence in situ hybridization.
